# Soil microbiome disruption reveals specific and general plant-bacterial relationships in three agroecosystem soils

**DOI:** 10.1371/journal.pone.0277529

**Published:** 2022-11-16

**Authors:** Michael J. DiLegge, Daniel K. Manter, Jorge M. Vivanco

**Affiliations:** 1 Center for Rhizosphere Biology, Department of Horticulture and Landscape Architecture, Colorado State University, Fort Collins, Colorado, United States of America; 2 USDA-ARS, Soil Management and Sugar Beet Research, Fort Collins, Colorado, United States of America; Universidade de Coimbra, PORTUGAL

## Abstract

Soil microbiome disruption methods are regularly used to reduce populations of microbial pathogens, often resulting in increased crop growth. However, little is known about the effect of soil microbiome disruption on non-pathogenic members of the soil microbiome. Here, we applied soil microbiome disruption in the form of moist-heat sterilization (autoclaving) to reduce populations of naturally occurring soil microbiota. The disruption was applied to analyze bacterial community rearrangement mediated by four crops (corn, beet, lettuce, and tomato) grown in three historically distinct agroecosystem soils (conventional, organic, and diseased). Applying the soil disruption enhanced plant influence on rhizosphere bacterial colonization, and significantly different bacterial communities were detected between the tested crops. Furthermore, bacterial genera showed significant abundance increases in ways both unique-to and shared-by each tested crop. As an example, corn uniquely promoted abundances of *Pseudomonas* and *Sporocytophaga*, regardless of the disrupted soil in which it was grown. Whereas the promotion of *Bosea*, *Dyadobacter* and *Luteoliobacter* was shared by all four crops when grown in disrupted soils. In summary, soil disruption followed by crop introduction amplified the plant colonization of potential beneficial bacterial genera in the rhizosphere.

## Introduction

Relationships between crop plants and their native bacterial communities have become increasingly important factors of crop production in agriculture. Recent discoveries have revealed positive correlations between soil microbial diversity and plant health, yield, disease suppression, and soil ecosystem cycling [1, 2]. Plants actively recruit their root microbiota by changing exudation patterns throughout growth to accommodate their developmental needs [3, 4]. Thus, cultivation of plants in microbially diverse soils likely provides an assorted selection pool of symbionts that could assist plant growth [5]. In contrast, several monocultured agroecosystems are express signs of soil microbial dysbiosis, or imbalances in the rhizosphere microbiota [6–11]. Such symptoms include reduced bacterial diversity [12] and/or higher plant pathogen abundance [6, 7, 13]. Further, studies suggest that annual cultivation of the same plant species could allow for a sense of “microbial habituation” to the presence of plants repeatedly grown in the same sites; thus, paving the way for imbalances to occur in soil microbial communities because of the unchanged host [14]. However, rhizospheric microbial rearrangements are not solely attributed to plant rhizodeposition, as the structure and function of soil microbiota has also been observed to shift in response to management practices, resulting in variable effects on plant health [9, 15, 16].

Recent attempts to reshape imbalances in agricultural microbial communities have shown that applying soil sterilization methods (via moist- or dry-heat, chemical fumigation, microwave, or gamma-irradiation, etc.) can eventually provide a more balanced rhizosphere microbiome and promote plant growth [17–19]. Other attempts to reduce the plant-health impacts of imbalanced soil microbial communities have used inoculations of plant-growth promoting rhizobacteria (PGPRs) [20]. Such studies often result in the inability of the inoculated microbes to establish, as native microbiota are habituated and more fit to maintain colonization of the soil/rhizosphere ecosystem [21, 22]. As an example, it was shown that when carbendazim (a fungicide) was used to disrupt soils prior to PGPR inoculation, the inoculated PGPR-microbes were able to show increases in colonization compared to the same microbes applied to undisrupted soils [21]. Additionally, a recent study shows that applying moist heat sterilization to soils reduced microbial load, subsequently allowing plants to recruit distinct microbiota from the native community, along with several plant growth-promoting microbial functions [17].

In the current study we explore plant rhizosphere communities that develop after soil disruption, and results show the presence of several plant-beneficial bacteria across different crops and soil types. Additional aims were to observe how plants recruit rhizobacteria from soils with differing managerial history and diversity in response to microbiome disruption and crop growth. To achieve these aims, we disrupted soils from three distinct agroecosystems (organic, conventional, and diseased) via autoclaving. Following disruption, crops from four differing families (Poaceae, Amaranthaceae, Asteraceae, and Solanaceae) were planted in disrupted and undisrupted counterparts of the same soil and grown in a greenhouse experiment. Rhizosphere samples were collected from plants grown in each soil after the experiment, and bacterial communities were analyzed by Illumina MiSeq sequencing of the bacterial 16S gene (V3-V4 region).

## Methods

### Soil collection, treatment, and chemical analysis

Soils used in this study were sourced from three agricultural ecosystems. Soil types, plants present during soil collection as well as geographical location of each site are as follows: (i) a USDA-certified organic cover crop field [Agricultural Research, Development and Education Center (ARDEC)-South, Specialty Crops Program, Colorado State University (CSU), Fort Collins, CO] growing mixed cover crop species (*Avena sativa* and *Vicia villosa*) and certified as USDA organic since 2003, referred to as “organic soil”; (ii) a USDA ARS no-till cultivation system (ARDEC-North, USDA-ARS, Fort Collins, CO) growing corn (*Zea mays*) annually supplemented 180 lbs/acre of nitrogen and referred to as “conventional soil” and; (iii) a 10-year old peach orchard (*Prunus persica*, cv. ‘Cresthaven’ grafted onto ‘Lovell’ rootstock; Western Colorado Research Center, Orchard Mesa, Grand Junction, CO) that is symptomatic of peach replanting disease and as such is referred to as “diseased soil”. Clean shovels were used to collect bulk and rhizosphere soil (< 40 cm depth) from the organic site after the removal of oat and hairy vetch plants. Thus, the rhizosphere soils of oat and vetch plants were included in the soil collection. Soils from the conventional site were collected like the organic site, but corn plants were growing during collection and corn’s rhizosphere soils were included. Soils (< 50 cm depth) from the diseased site were collected nearby peach tree trunks to represent the peach tree rhizosphere while also being non-destructive to the orchard.

During soil sampling at each location, all soils were collected and stored in coolers with ice packs and transported to the lab for subsequent experimentation. The soils were sifted through an 8 mm soil sieve to remove large debris. Sub-samples of each field soil type (organic, conventional, and diseased) were collected, air dried to 30–40% moisture, and stored at -20°C for later DNA extraction. After sieving large debris out of each soil type, soils from each field were divided into two equal parts. The first part of each soil experienced no further treatment and is referred to as undisrupted soil. The second part was exposed to steam sterilization using a Steris brand autoclave for three 15-minute liquid cycles at 121 °C and is referred to as the disrupted soil treatment. Standard chemical analyses of all soils were conducted by the Soil, Water and Plant Testing Laboratory at Colorado State University in groups of two replicates per treatment to determine the changes in composition brought upon by autoclaving. Parameters determined were as follows: pH, electrical conductivity, lime estimate, percent organic matter, soil texture and the following nutrient availabilities were analyzed (in ppm): NO_3_-N, P, K, Zn, Fe, Mn, and Cu.

### Plant growth experiment

The disrupted and undisrupted soils from each site were poured into individual plastic pots (~400 g per pot, pot size: 7 x 10 x 8.5 cm). For each soil treatment, seeds of corn, beet, lettuce, and tomato were surface sterilized with 3.0% NaClO, rinsed three times with sterile water, and imbibed in sterile water for 24 hours prior to planting. After imbibition seeds were sown into pots at a rate of 3 seeds per pot (with 10 pots per crop per soil treatment; total n = 300). After the emergence of one or more seedlings per pot, extra seedlings were removed to allow for 10 uniform replicates (at a rate of one plant per pot) within a treatment, and then the 7-week growth period began. Additionally, in all treatments, both disrupted and undisrupted soil samples were placed into pots in the absence of plants and included in the experimental block design. These no plant controls (NPCK) provided insights to soil bacterial re-arrangements without the plant inputs. The experiment was conducted at CSU’s Horticulture Center Greenhouse Facility under an average temperature of 26.26 ± 2.17 °C (79.3 ± 3.9 °F) and relative humidity of 38.3 ± 14.5%. To avoid accidental transplanting of soil particles (containing microbes) from differing ecosystems via splashing during irrigation, the experimental replicates were randomized within their respective soil block treatment (i.e. crops in disrupted organic soils were randomized within the disrupted organic soil block). All plants received the same volume of water during irrigation, and plants received no fertilizer during the experiment based on sufficient nutrient content of each soil. Experimental plants were allowed to grow for 7 weeks until the growing period was concluded, and plant growth parameters were recorded.

### Plant experiment—Data collection

After the 7^th^ week of growth, plants were harvested, cut at the root-shoot axis and the above-, below- and total fresh-weight biomass measurements of each replicate were recorded. On the same day, rhizosphere soils were collected via gentle brushing of plant roots overtop Ziploc bags to remove any root-adhering soil. Of the ten plant replicates in each treatment, two rhizosphere soil samples (or soil-core samples from the NPCKs, depth: 2–5 cm) were combined for a total of five soil or rhizosphere microbiome samples to represent each experimental treatment. Soil samples were air dried, transported to the lab and stored at -20 °C until subsequent DNA extraction. Following fresh-weight biomass measurements and rhizosphere soil collection, plants were placed into individual paper bags, dried in an oven for 48 h at 65 °C, and then the dry-weight biomass was recorded. The mean biomass measurements (both fresh and dry) from each treatment were compared with a two-way ANOVA model using Prism’s GraphPad (Vers. 8.2.1) and pairwise comparisons were conducted using Siddak’s multiple comparison tests (GraphPad Software, La Jolla California, USA).

### Soil DNA extraction and bacterial community analysis

Total genomic DNA (gDNA) was extracted from 0.25 grams from three replicates of soil and rhizosphere samples from each experimental treatment. Samples were extracted using the E.Z.N.A Soil DNA Kit (Omega Bio-Tek, Norcross, GA, USA) according to the manufacturer’s instructions. Following extraction, nucleic acid concentration and sample purity were quantified and determined using NanoDrop 2000 Spectrophotometer (Thermofisher, Waltham, MA, USA). gDNA samples were then stored at −20 °C prior to Illumina MiSeq library preparation and downstream microbiome analyses.

### Microbiome analyses

#### Quantification of bacterial cells from gDNA

16S rRNA copies per gram of soil were determined by performing quantitative Polymerase Chain Reaction (qPCR) of all gDNA samples against a standard curve of purified *Pseudomonas putida* KT2440 16s gDNA. The qPCR conditions were as follows: aliquots of each quantified gDNA sample were collected to prepare template DNA at a concentration of 5 ng mL^-1^. The qPCR reactions were performed in 10 mL reaction volumes containing 1 mL of template DNA and 9 mL of the master mix. The master mix consisted of 5 μL SYBR Green (QuantaBio, Beverly, MA, USA), 0.5 mL of each forward and reverse primer (10 mM) and brought to a total volume of 9 mL using 3 mL of molecular grade water. The qPCR thermal cycling conditions for bacterial quantification were as follows: 95°C for 8 minutes and 30 seconds, 30 amplification cycles (95°C for 15 seconds, 58°C for 30 seconds, 72°C for 60 seconds) followed by a final annealing stage at 72 °C for 5 minutes. The mean 16s rRNA copies from each crop treatment were compared to their respective NPCK (disrupted or undisrupted) by using a two-way ANOVA model with R Studio’s aov function [23].

#### Library preparation for Illumina MiSeq sequencing

The initial soil gDNA samples were diluted at the ratio of 1:5 with molecular water to reduce PCR inhibitors introduced during DNA extraction. Another round of PCR targeting the V3-V4 region of the bacterial 16S rRNA gene was performed using the primer set 341f/806rb (341f: 5′– TCGTCGGCAGCGTCAGATGTGTATAAGAGACAGCCTACGGGAGGCAGCAG-3′. 806rb: 5′- GTCTCGTGGGCTCGGAGATGTGTATAAGAGACAGGACTACHVGGGTATCTAATCC-3′) to target bacterial 16s rRNA and to attach Illumina MiSeq adapters, denoted via underlining in the above primer sequences [24]. This second round of PCR was performed in 20 μL reaction volumes containing 2 μL of template DNA and 18 μL of the master mix. The master mix consisted of 10 μL 2X Maxima SYBR Green (Thermo Scientific, Waltham, MA, USA), and 2 μL each (10 μM) of forward and reverse primers and brought to a total volume of 18 μL using 4 µL of molecular grade water. The PCR thermal cycling conditions were as follows: 95°C for 5 minutes, 30 amplification cycles (95°C for 40 seconds, 55°C for 120 seconds, 72°C for 60 seconds) followed by a final annealing stage at 72°C for 7 minutes to reduce chimeric reads. A standard curve using purified *Psuedomonas putida* KT2440 gDNA was run with the samples to quantify the starting rRNA copies per g^-1^ soil. Resulting amplicons were then purified using an in-house preparation of solid phase reversible immobilization (SPRI) magnetic beads based on a modified protocol of [25] and original protocol of [26].

A second PCR cycle was then conducted to attach unique Illumina Nextera XT indices to each bead cleaned sample for subsequent sample demultiplexing. Each well contained 5 µL of first round and bead-cleaned qPCR product, 25 µL of 2X Maxima SYBR Green (Thermo Fisher Scientific, Waltham, MA, USA), 5 µL each of both forward and reverse indices were combined along with 10 µL of water, bringing the total volume to 50 µL. PCR conditions were as follows: 95°C for 3 minutes, 8 amplification cycles (95°C for 30 seconds, 55°C for 30 seconds and 72°C for 30 seconds) followed by final annealing of 72°C hold for 5 minutes. The resulting PCR product was again SPRI-bead cleaned using the same methods previously mentioned. Amplicons were then quantified using a Qubit fluorometer (Thermo Scientific, Waltham, MA, USA) prior to normalization and pooling. The final pool was run on a TapeStation system (Agilent Technologies, Santa Clara, CA, USA) to determine size and purity of amplicons, and Kapa Biosystems (Sigma-Aldrich, St Louis, MO, USA) qPCR was performed according to the manufacturers’ instructions to determine concentration. The final pooled sample was diluted to 4 nM and the DNA library was denatured with 0.2 N NaOH, diluted to 10 pM using provided HT1 buffer, and spiked with 20% PhiX library standard diversity-control. Illumina’s MiSeq v3 600-cycle Reagent Kit (Illumina, San Diego, USA) was used for library dilution and loading onto the MiSeq at CSU’s Next Generation Sequencing Laboratory (Fort Collins, CO).

#### Bacterial 16s rRNA gene sequence analysis

De-multiplexed raw fastq files were processed with the DADA2 pipeline using R Studio’s Bioconductor packages [27]. Briefly, all primers were removed from each sequence using the open-source Python program Cutadapt [28] and amplicon sequence variants (ASVs) were inferred using the default pipeline in DADA2, and low abundant ASVs (≤1) were filtered from the dataset. Each sequence variant identified in DADA2 was classified to the closest reference sequence contained within the Silva reference database (Version 132) using DADA2’s naïve Bayesian classifier. Each taxonomic profile assigned was used to determine bacterial genus level abundance values. Downstream analyses were conducted using R Studio’s Phyloseq and vegan packages or myPhyloDB (https://myphylodb.scinet.usda.gov/myPhyloDB/home/) [29–32]. Samples were rarified at a cutoff of 7500 reads using myPhyloDB prior to downstream analysis applications using myPhyloDB or R Studio. All replicate samples within one treatment were aggregated prior to downstream analyses, and analyses were conducted on classified ASVs aggregated at the lowest classifiable taxonomic level of interest (i.e., phylotypes). Measurements of alpha-diversity assigned to treatments were determined using the Shannon diversity index, as this diversity measure accounts for both richness and evenness within each sample. A two-way ANOVA model was applied to compare mean alpha-diversity values from each disrupted crop treatment to their respective undisrupted crop treatment. Additionally, disrupted crop treatments were compared to the disrupted NPCK treatments with R Studio’s Analysis of Variance package [24]. R Studio’s vegan package was used with the Bray-Curtis dissimilarity index to quantify differences in community composition and beta diversity between samples [30]. Distances were visualized using principal coordinates analyses (PCoA) created using Prism’s GraphPad (Vers 8.2.1, GraphPad Software, La Jolla California, USA). The myPhyloDB software was used to perform a complementary non-parametric multivariate statistical test, including permutational analysis of variance (perMANOVA) as well as differential abundance analyses (FDR < 0.1) to test for differences in microbial communities between treatments [31]. Differential abundances were determined using a negative binomial GLM (DiffAbund function) in myPhyloDB (v.1.2.0). A Venn diagram of these data was constructed using the jvenn software [32].

## Results

### Effect of soil disruption on agroecosystem soil nutrients

To determine the effect of autoclave disruption on soils, soil pH, electrical conductivity, lime estimate, percent organic matter, texture, and parts per-million (ppm) of the following nutrients: nitrate-nitrogen, phosphorous, potassium, zinc, iron, manganese, and copper were measured. These parameters were analyzed for all disrupted and undisrupted soil samples (Table 1). Across all soil types, phosphorous and manganese were significantly increased after autoclave disruption. For organic soil, P and Mn were increased by 0.299-fold (p-value 0.004) and 2.292-fold (p-value: < 0.001), respectively (Table 1). For conventional soil, P and Mn were increased by 0.462-fold (p-value: 0.002) and 1.792-fold (p-value: < 0.001), respectively (Table 1). For diseased soil, P and Mn were increased by 0.241-fold (p-value: 0.016), and 7.571-fold (p-values: < 0.001), respectively (Table 1). Interestingly, only the diseased agroecosystem soil experienced significant reductions in the availability of both zinc (0.137-fold, p-value: 0.002) and copper (0.318-fold, p-value: < 0.001) after autoclaving. Neither organic nor conventional soil experienced significant reductions in nutrient availability after autoclave disruption (Table 1).

**Table 1:** Chemical analyses of soils (n = 2 / agroecosystem) tested. Green- or red-highlighted cells in the p-value column (within each larger agroecosystem column) indicate significant increases or decreases in the parameter tested as a result of applying disruption to the soils.

(n = 2)	Organic Soil			Conventional Soil			Diseased Soil		
	Undisrupted	Disrupted	p-value	Undisrupted	Disrupted	p-value	Undisrupted	Disrupted	p-value
**pH**	7.62	7.745	0.341	7.985	8.175	0.094	7.82	7.93	0.448
**EC (mmhos/cm)**	2.45	2.58	0.909	0.6	0.7	0.966	0.615	0.71	0.973
**Lime Estimate**	high	med/high	-	high	high	-	high	high	-
**% Organic Matter**	2.85	2.25	0.48	2.75	2.85	0.999	2.5	2.3	0.984
**NO3-N ppm**	24.15	23.8	0.991	7.35	6.45	0.709	0.65	0.8	1
**P ppm**	17.75	22.4	0.004	11.25	16.45	0.002	14.35	17.8	0.016
**K ppm**	566.5	525	0.072	465	437.5	0.282	254.5	240.5	0.811
**Zn ppm**	1.4	1.2	0.178	1.15	0.9	0.081	4	3.45	0.002
**Fe ppm**	7.6	7.55	1	5.55	7.35	0.302	13.3	10.45	0.066
**Mn ppm**	12	39.5	0	1.85	35	0	4.2	36	0
**Cu ppm**	3.7	3.35	0.233	2.3	2.1	0.689	7.7	5.25	0
**Texture**	Silty Clay / Clay	Silty Clay	-	Silty clay / clay	Silty Clay	-	Sandy clay / SC loam	Sandy Clay	-

### Effect of soil disruption on plant biomass

**Fig 1:**
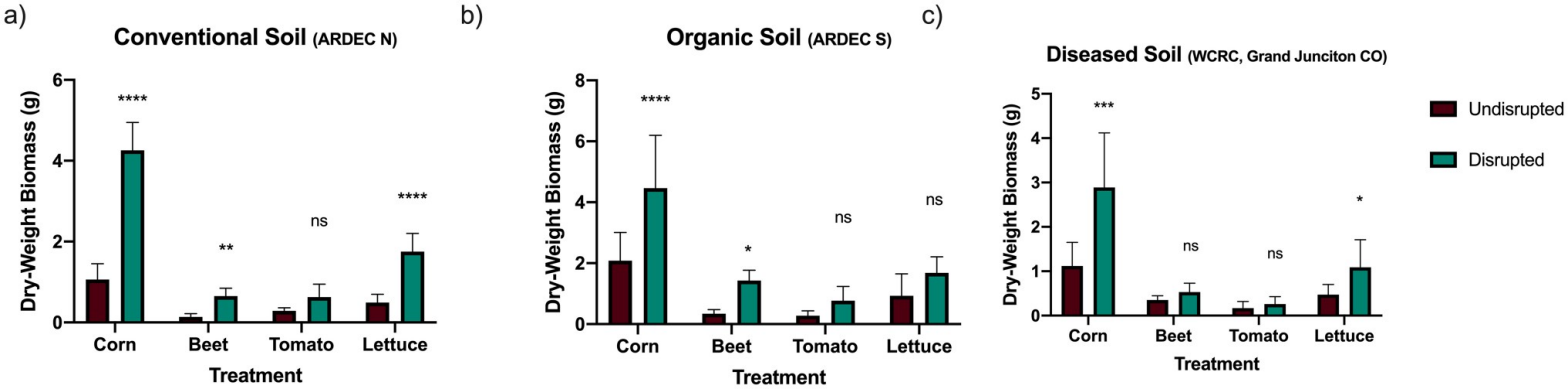
Mean dry-weight (DW) biomass measurements for each crop in each soil treatment (n = 10 per crop per soil treatment, ± standard deviation). (a) DW biomass results from crops grown in organic soil (b) DW biomass of crops grown in conventional soil (c) DW biomass of all crops grown in diseased soil. Red or blue bars represent the mean DW biomass for each crop in undisrupted or disrupted soil respectively. Significant differences between mean. DW biomass of crops grown in disrupted or undisrupted soils are denoted by “*”, “**”, “***” or “****”. Figure created using GraphPad’s Prism (Vers. 8.2.1).

Above and below ground measurements of plant dry-weight (DW) biomass were summed to analyze disruption effects on total DW biomass of crops (Fig 1). The total DW biomass of corn was significantly increased when compared to total DW of corn grown in soils with no disruption (p-values: <0.001, <0.001, and <0.001 for organic, conventional, and diseased; respectively). The total DW biomass of beet was significantly promoted when grown in disrupted organic and disrupted conventional soils (p-values: 0.012 and 0.008, respectively), but not in the diseased soil (p-value: 0.914). Total DW biomass of tomato was not significantly altered after growth in any of the disrupted soils (p-values in organic, conventional and diseased were 0.530, 0.1503, and 0.993, respectively). Lastly, the total DW of lettuce was significantly increased after growth in disrupted conventional and disrupted diseased soils (p-values: < 0.0001 and 0.04, respectively), but not in the disrupted organic soil (p-value: 0.1451). The average gain in DW biomass, fresh weight biomass bar graphs, and percent increases observed for each treatment are available as supplementary data (S1 Fig, S1 and S2 Tables).

### Microbiome analyses

#### Rhizobacterial cell quantification following soil disruption

**Fig 2:**
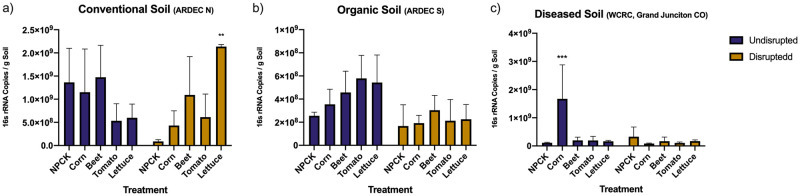
Mean 16s rRNA copies per gram of fresh weight of each soil (n = 3 per crop per soil treatment, ± standard deviation). (a) 16s rRNA copies resulting after crop growth in organic soil (b) 16s rRNA copies resulting after crop growth in conventional soil (c) 16s rRNA copies resulting after crop growth in diseased soil. Purple or yellow bars represent the mean number of 16s rRNA copies for each crop grown in undisrupted or disrupted soils, respectively. Significant differences in 16s rRNA copies between the NPCKs and crop treatments are denoted by “*”. Figure created using GraphPad’s Prism (Vers. 8.2.1).

For all soil gDNA samples, the final concentration of nucleic acids ranged from 15.2 ± 8.6 ng / uL after extraction. For qPCR analysis, 16s rRNA copies per gram of soil from the no plant control samples (NPCKs) for each agroecosystem (disrupted or undisrupted) were compared to 16s rRNA copies from crop samples to see changes in bacterial cells resulting from crop growth in disrupted or undisrupted soils. In organic soils, our qPCR analysis shows no significant differences observed between soils with crops compared to the NPCKs, regardless of disruption (Fig 2A). For conventional soils, no significant differences in 16s rRNA copies were observed between the undisrupted NPCKs and the undisrupted soil that grew crops. After disruption of the conventional soils, the lettuce rhizosphere significantly promoted 16s rRNA copies compared to the disrupted conventional NPCK (p-value: 0.011, Fig 2B). In diseased soil, the growth of corn in undisrupted soil increased 16s rRNA copies, compared to results from the undisrupted diseased NPCK (p-value: 0.004, Fig 2C). Otherwise, applying disruption to remaining diseased soil samples did not cause significant differences in 16s rRNA copies between treatments and the NPCKs (Fig 2C).

Interestingly, the NPCKs provided insights into soil bacterial rearrangements occurring in the absence of plants following disruption. In both the organic and conventional soils, soil bacterial trends followed those observed when crops were introduced to these systems after disruption (e.g., 16s rRNA copies were lessened after disruption compared to their undisrupted counterparts). However, in the diseased soil 16s rRNA copies from the disrupted NPCKs were not significantly different form the undisrupted soils (p-value: 0.975). The associated statistical analysis for these data can be visualized in S3 Table.

#### Illumina MiSeq results of rhizosphere and soil samples

After filtering reads, removing singleton ASVs and rarifying, Illumina MiSeq paired-end sequencing generated a total of 4,264,022 reads resulting in an average of 46,348 reads per sample. Seven out of 104 total samples did not meet rarefaction criteria (min. 7,500 reads) and were dropped. Bacterial diversity resulting from crop growth in disrupted soils were compared to their disrupted NPCKs within the same agroecosystem. Additional comparisons were made from the disrupted crop growth treatments to their crop-counterparts grown in the undisrupted parts of the same soil. Bacterial diversity (Shannon index) was significantly different between communities from the disrupted and undisrupted corn grown in the diseased agroecosystem (p-value 0.041), as well as from the disrupted and undisrupted lettuce grow in the organic agroecosystem (p-value: 0.023) but not for any other plant/soil combination (Table 2, S4 Table).

**Table 2:** Shannon’s diversity index. Shannon’s diversity index values were recorded for all samples and determined using the R’s vegan package (Vers. 25–6). (NPCK = No plant controls).

Soil Type	Treatment	Crops				
		Corn	Beet	Tomato	Lettuce	NPCK
**Organic**	Field Collected	2.4				
	Undisrupted	4.31	4.64	4.5	3.88	4.48
	Disrupted	4.44	4.41	4.63	4.66	4.65
	p-value	0.999	0.943	0.999	*0.023	0.989
**Conventional**	Field Collected	4.63				
	Undisrupted	3.98	4.4	4.44	4.38	4.36
	Disrupted	4.44	4.14	4.16	4.22	3.82
	p-value	0.886	0.981	0.969	0.999	0.354
**Diseased**	Field Collected	4.46				
	Undisrupted	5	4.41	4.99	4.63	4.4
	Disrupted	4.15	4.19	4.28	4.16	3.63
	p-value	*0.041	0.991	0.125	0.608	0.079

#### Principle coordinate analysis of rhizobacterial community rearrangements following soil disruption

**Fig 3:**
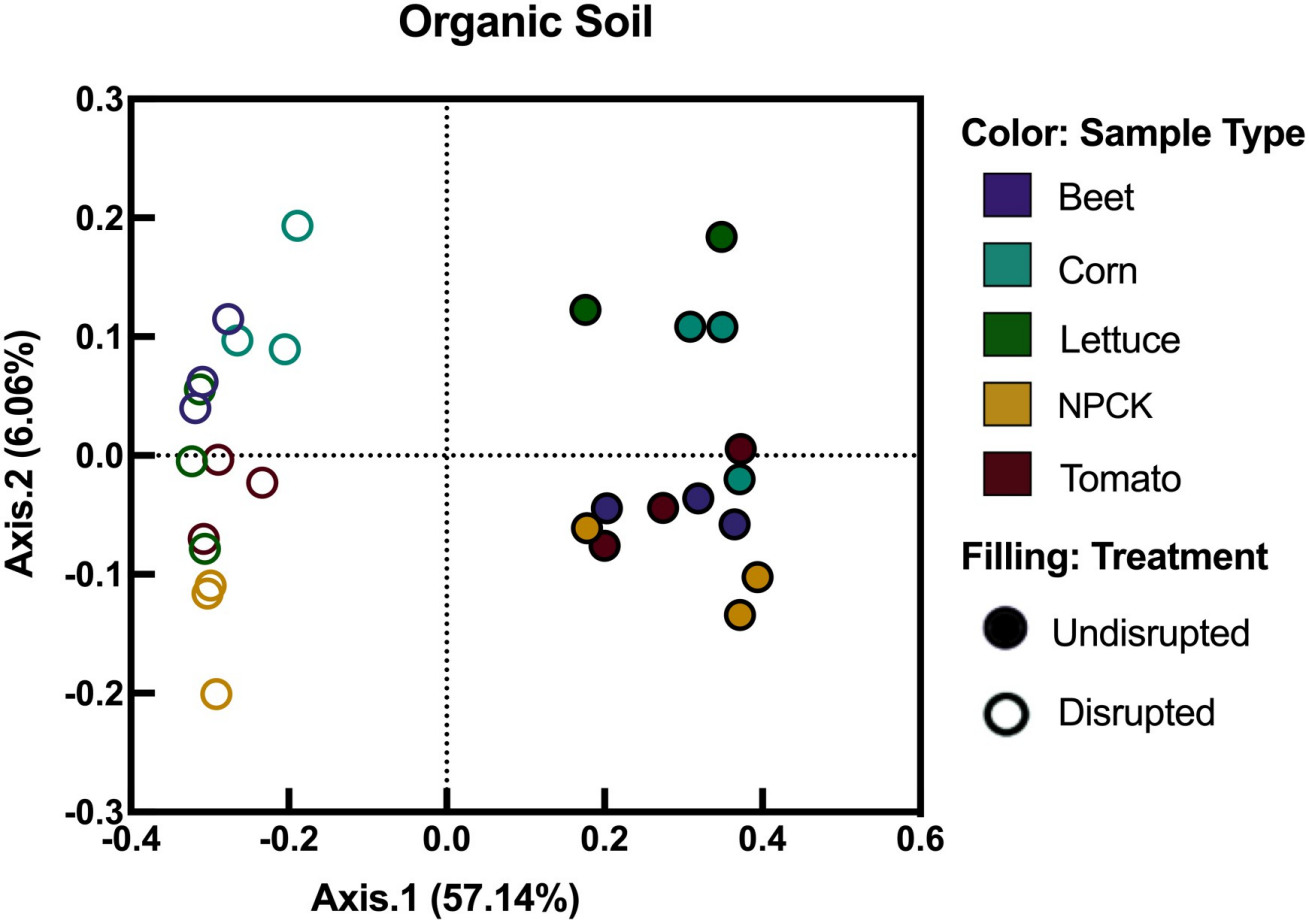
Principal coordinate analyses (PCoA) of rhizobacterial communities after crop growth in undisrupted and disrupted organic agroecosystem soils. Colored circles indicate samples treated with disruption, whereas hollow circles indicate undisrupted samples, respectively. Purple, teal, green, yellow, or red color represents rhizospheric samples from beet, corn, lettuce, NPCKs or tomato samples, respectively. Figure created using GraphPad’s Prism (Vers. 8.2.1).

In the organic soils, a permutational analysis of variance was used to measure differences in bacterial communities due to soil disruption (p-value: 0.001, R^2^: 0.549, Axis.1: 56.14%). Furthermore, our analyses revealed that each crop family was able to recruit significantly different rhizobacteria independent of disruption (p-value: 0.001, R^2^: 0.1372, Axis.2: 6.06%) (Fig 3). A beta-diversity analysis using betadisper further demonstrates that reads resulting in crop roots from the disrupted organic soils had significantly higher dispersion than rhizobacterial reads from the same crops grown in undisrupted organic soil (p-value 0.027).

**Fig 4:**
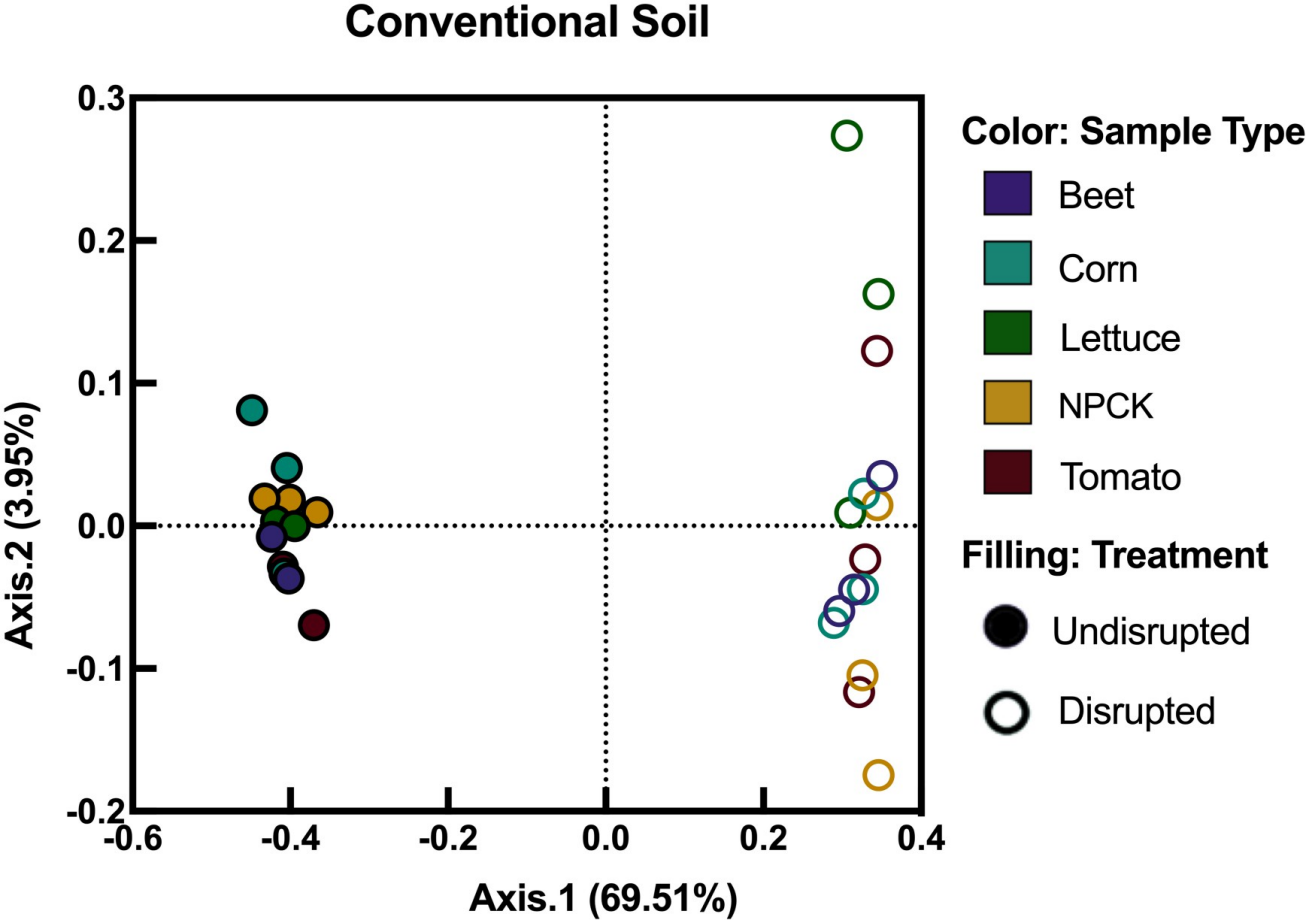
Principal coordinate analyses (PcoA) of rhizobacterial communities after crop growth in undisrupted and disrupted conventional agroecosystem soils. Colored circles indicate samples treated with disruption, whereas hollow circles indicate undisrupted soils, respectively. Purple, teal, green, yellow, or red color represents rhizospheric samples from beet, corn, lettuce, NPCKs or tomato samples, respectively. Figure created using GraphPad’s Prism (Vers. 8.2.1).

In the conventional soil, our analyses showed that disruption produced the strongest significant differences in bacterial community composition (p-value: 0.001, R^2^: 0.693, Axis.1: 69.59%) when comparing reads from disrupted to the undisrupted conventional soil. Furthermore, crops grown in conventional soil were able to recruit significantly different rhizobacteria by plant-family, whether or not disruption occurred (p-value: 0.001, R^2^: 0.060, Axis.2: 3.95%) (Fig 4). Our beta-diversity analysis also showed that once disrupted, rhizobacterial reads in crops roots showed significantly higher dispersion compared to the rhizobacterial reads from their same-crop counterpart grown in undisrupted conventional soil (p-value 0.015) (Fig 4).

**Fig 5:**
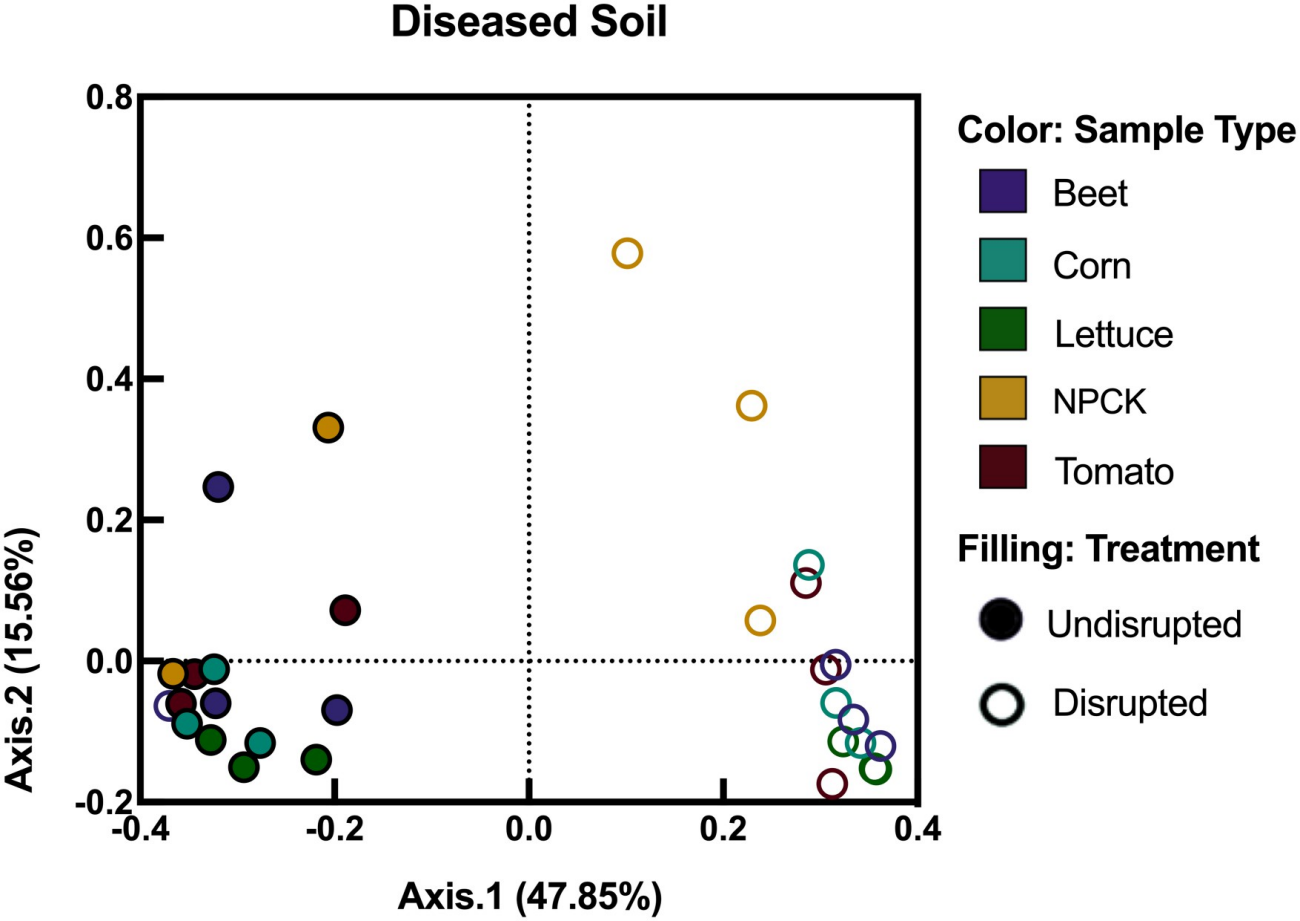
Principal coordinate analyses (PcoA) of rhizobacterial communities after crop growth in undisrupted and disrupted diseased agroecosystem soils. Colored circles indicate samples treated with disruption, whereas hollow circles indicate undisrupted soils, respectively. Purple, teal, green, yellow, or red color represents rhizospheric samples from beet, corn, lettuce, NPCKs or tomato samples, respectively. Figure created using GraphPad’s Prism (Vers. 8.2.1).

The diseased soil demonstrated the weakest effect regarding microbiome disruption. Again, soil disruption resulted in significantly different bacterial communities when comparing reads from the disrupted to undisrupted diseased soil (p-value: 0.001, R^2^: 0.461, Axis.1: 47.85%). Still, in diseased soils each individual crop family recruited significantly different rhizobacteria from other crop families regardless of disruption (p-value: 0.001, R^2^: 0.148, Axis.2: 15.56%) (Fig 5). The beta-diversity analysis for the disrupted and undisrupted diseased soil samples shows that rhizobacteria in disrupted crop roots resulted in similar dispersion as the rhizobacterial reads from their counterpart (i.e. the same crop type in undisrupted diseased soil) (p-value 0.08).

#### Rhizobacteria recruited by crops vs no plant controls (NPCKs) in undisrupted and disrupted soils

In both undisrupted and disrupted treatments, bacterial reads were pooled across all three soil agroecosystems and grouped by crop type. The abundances of these bacteria from each crop were then compared to the respective pooled NPCK samples (undisrupted or disrupted) to properly attribute abundance alterations to plant presence. Differential abundance analyses revealed significantly altered bacterial genera resulting from crop presence (Tables 3 and 4). Across the pooled undisrupted treatments, four bacterial genera were significantly altered in abundance by crop presence (Table 3), whereas twelve bacterial genera were altered in abundance when crops were grown in the pooled disrupted soils (Table 4).

**Table 3:** Shared and unique significant abundance increases of genus-level bacteria, prompted by each crop in all undisrupted agroecosystems (FDR: False discovery rate). Gray highlighting indicates the genus was not significantly altered within the crop rhizosphere).

Genus	No-Plant basemean	Beet basemean	p-value	FDR	No-Plant basemean	Corn basemean	p-value	FDR	No-Plant basemean	Lettuce basemean	p-value	FDR	No-Plant basemean	Tomato basemean	p-value	FDR
*Azohydromonas*					101.401	50.701	< 0.001	0								
*Catellatospora*									0	11.543	< 0.001	0.071				
*Pseudomonas*									9.969	177.193	< 0.001	0.071				
*Rhizorhapis*									0	31.76	< 0.001	0				

**Table 4:** Shared and unique significant abundance increases of genus-level bacteria, prompted by each crop in all disrupted agroecosystems (FDR: False discovery rate). Gray highlighting indicates the genus was not significantly altered within the crop rhizosphere.

Genus	No-Plant basemean	Beet basemean	p-value	FDR	No-Plant basemean	Corn basemean	p-value	FDR	No-Plant basemean	Lettuce basemean	p-value	FDR	No-Plant basemean	Tomato basemean	p-value	FDR
*Dyadobacter*	0	9.726	< 0.001	0.1	0	16.783	< 0.001	0.041	0	54.051	< 0.001	0				
*Lacibacter*									0	7.47	< 0.001	0.046				
*MM2*									0	8.532	< 0.001	0.046				
*Neorhizobium*									0	9.853	< 0.001	0.01				
*Niastella*									2.757	50.557	< 0.001	0.046				
*Novosphingobium*	15.54	313.827	< 0.001	0.03												
*Oscillatoria_PCC-6304*					57.927	0.3	< 0.001	0.041								
*Qipengyuania*									150.482	12.853	< 0.001	0.046				
*Rhizorhapis*									0	32.106	< 0.001	0				
*Rhodobacter*					43.719	0	< 0.001	0.041	43.719	0	< 0.001	0.036				
*Sphingobium*													2.074	113.283	< 0.001	0.005
*Verrucomicrobium*									0	23.053	< 0.001	0.046				

#### Differences in bacterial recruitment by the same crops in undisrupted vs disrupted soils

The ability for crops to alter abundance of bacterial genera during growth in the disrupted soils, compared To their same-crop counterpart in undisrupted soils, was also of interest. Across all disrupted soils, corn growth significantly increased abundances of 29 bacterial reads compared to corn growth in undisrupted soil (S5 Table). Beet plants grown in disrupted soils significantly increased abundances of 16 bacterial reads (S6 Table). Lettuce growth in the disrupted soils significantly increased abundances of 29 bacterial reads (S7 Table). Lastly, tomato growth in disrupted soils significantly increased abundances of 20 bacterial reads (S8 Table). The bacterial genera increased in the disrupted no plant controls were reported (S9 Table) and these genera were filtered out from the crop tables (S5–S8 Tables) to attribute abundance increases to plant presence rather than soil-irrigation or greenhouse influence.

#### Shared bacterial abundance increases among crops following soil disruption

**Fig 6:**
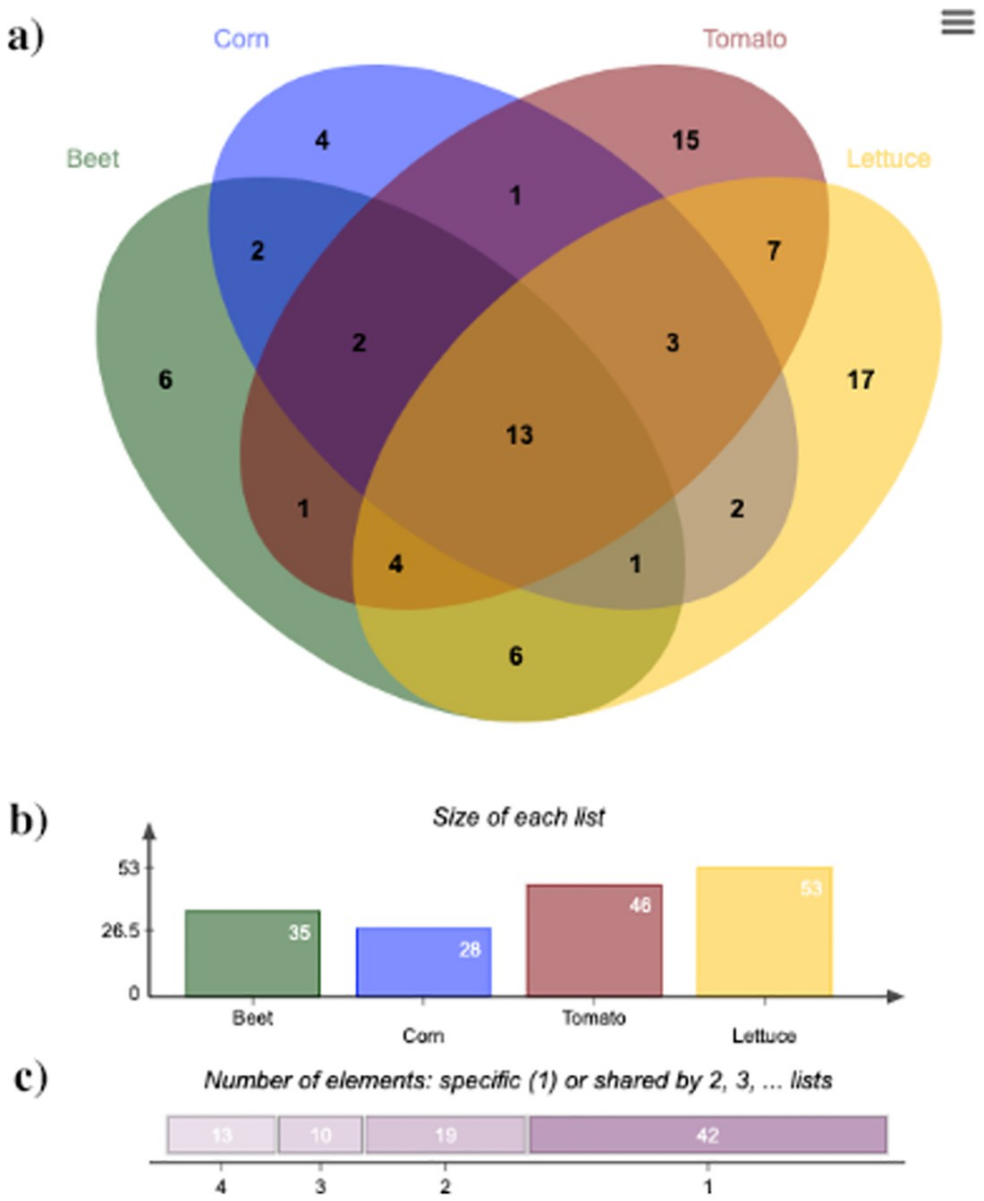
Venn diagram displaying the shared and unique bacterial reads from pooled crop rhizospheres after disruption. The list of full crop-shared or crop specific bacterial reads can be visualized in S10 Table. (A) Venn diagrams noting the number of shared or specific genera for each crop (green = beet, blue = corn, red = tomato, yellow = lettuce). (B) Bar graph showing the size of bacterial increased in abundance by each crop after disruption. (C) Display showing the number of bacterial reads unique to one (42) or shared by two, three or four crops (19, 10, 13, respectively). Figure created using jvenn (2007).

Across all disrupted soils, growth of corn, beet, lettuce, and tomato significantly increased abundances of several unique and overlapping bacterial reads (Fig 6, S10 Table). However, if bacterial reads were observed to increase in the disrupted NPCKs, these were filtered out and were assumed to be bacteria able to grow in the absence of a plant (S9 Table). Across all disrupted agroecosystems, all four crops significantly promoted the abundance of eleven bacterial genera, and two bacterial families (Fig 6, S10 Table). A total of nine other bacterial genus level reads were significantly increased in abundance and shared by different combinations of three out of the four crops tested. Corn, lettuce, and tomato growth increased the abundance of bacterial families Cellvibrionaceae, Fibrobacteraceae, and KD3-93. Beet, tomato, and lettuce increased the genera *Algoriphagus*, *Devosia*, *Oligoflexus* and *Articibacter*. Beet, corn, and tomato growth increased abundances of the family Devosiaceae and the genus *Opitutus* (Fig 6, S10 Table).

Several other bacterial reads were observed to be increased in abundance and shared in the rhizosphere by different combinations of only two out of the four crops. Bacterial genera increased by the growth of tomato and lettuce were *Anoxybacillus*, *Flavobacterium*, *PCC-7104* and *Verrucomicrobium*, in addition to the families Beijerinckiacea and Cyclobacteriaceae. Corn and lettuce growth promoted abundances of the bacterial orders Bacillales and Candidatus_Peribacteria. Corn and tomato growth increased abundances of the genus *Stenotrophomonas*. Beet and lettuce both increased abundances of the genera *Quadrisphaera*, *Brevibacillys*, *SHPL14*, and *Roseomicrobium*, in addition to the family Sphingomonadaceae and the order Sericytochromatia. Beet and tomato both promoted abundances of the genus *Ammoniphilus*, whereas the growth of both beet and corn promoted the genus *Caulobacter* and the order Microgenomatia (Fig 6, S10 Table).

#### Crop-specific bacterial abundance increases following soil disruption

There were also several crop-specific bacterial reads increased in abundance following soil disruption. For Example, corn growth significantly promoted abundances of two genera (*Sporocytophage* and *Pseudomonas*) along with two families (Fibrobacteraceae and Rhodothermaceae) (Fig 6, S10 Table). Growth of beet promoted abundances of six genera (*Roseococcus*, *Peredibacter*, *Flavisolibacter*, *Parasegetibacter*, *UTBCD1* and *Solimonas*) (Fig 6, S10 Table). Nine bacterial genera were increased in abundance in response to lettuce growth (*Rhodobastu*, *Roseomonas*, *Rhodoferax*, *Fluviicola*, *Cytophaga*, *Knoellia*, *PCC-6304*, *Tepidisphaera*, and *Brevifollis*). Lettuce growth also increased abundance of four bacterial families (Burkholderiaceae, Caulobacteraceae, Opitutaceae, and Sporolactobacillaceae) (Fig 6, S10 Table). The growth of tomato increased abundance of nine bacterial genera (*Bacillus*, *Chelatococcus*, *Asticcacaulis*, *Yongharparkia*, *Aminobacter*, *Cereibacter*, *Mucilaginibacter*, *Shimazuella*, and *Thermomonas*), three bacterial families (Archangiaceae, Hydrogenedensaceae, and Verrucomicrobiaceae) and two orders (Candidatus_Peribacteria and Candidatus_Pacebacteria) (Fig 6, S10 Table).

## Discussion

### Effects of autoclave disruption on agroecosystem soils

Distinct field management techniques (organic, conventional, or disease management-focused) indirectly condition native soil microbes to their hosts and management practices, and these changes in microbiota may positively or negatively impact plant growth [6, 7, 16, 18]. Here, we investigated how soil factors (both biological and chemical) in distinct cropping systems responded to autoclave disruption, and how these responses impact plant growth and rhizosphere bacteria. Soil autoclaving has been reported to increase extractable P and Mn content [33, 34] and this effect was observed in all soils in the present study. Interestingly and unique to the diseased soil, both Zn and Cu content were reduced after disruption. Similar to [17], plant growth promotions were observed for each crop when grown in disputed soils, compared the same crops grown in undisrupted soil. The increased plant growth effect is probably not a result of increased P and Mn content, but more likely due to soil disruption and the resulting rhizobacteria as influenced by crop growth. Our 16S qPCR analyses revealed variable changes on 16s rRNA copies resulting from disruption and crop growth, with most 16s rRNA copies being reduced compared to undisrupted samples. As such, resulting increases in plant growth may be due to i) reducing plant-microbe competition for soil resources, or ii) the reduced population of soil bacteria lessened bacteria-bacteria competition for plant deposited resources, favoring recruitment of plant growth promoting related taxa.

### Effects of autoclave soil disruption on rhizobacterial community rearrangements

Upon collection, it was observed that a higher bacterial diversity level (Shannon Index) persisted in the conventional and diseased soils compared to the organic site. However, disruption and plant growth resulted in the organic site to showing the highest bacterial diversity value out of all three soils. It was also observed that following growth in the disrupted soils, plants were able to significantly increase the abundances of a greater number of bacterial genera (12 genera) compared to their growth in undisrupted soils (4 genera). Combining the observations shared by disrupting three distinct soils (increases in plant growth, Shannon’s diversity index, and number of recruited genera following disruption); soil disruption may likely serve as a promising managerial technique in restoration of a balanced soil microbial community.

### Community analysis of rhizobacterial community rearrangements following soil disruption

Soil disruption also caused significantly different bacterial community composition from the undisrupted soils, across all sites [Bray Curtis distance, disruption p-values: 0.001 (for all soils)]. These findings demonstrate that each crop was able to recruit significantly different rhizobacteria, based on the soil the crop was grown in, and whether disruption was administered or not. The strongest effect of crop-specific recruitment is exemplified in the organic soil. Each crop was shown to promote distinct rhizobacteria, both when comparing the crop to its undisrupted counterpart, and when comparing rhizobacteria from differing crop types to one another when all were grown in disrupted or undisrupted soils (disruption effect p-value: 0.001, Axis.1: 57.14%; crop effect p-value: 0.001, Axis.2: 6.06%; R^2^: 0.1372).

Like the organic site, crops grown in conventional soil were also shown to recruit significantly different communities between different crops and between the same crops in disrupted or undisrupted soil (disruption effect p-value: 0.001, Axis.1: 69.51%; crop effect p-value: 0.001, Axis.2: 3.95%; R^2^: 0.060). Conventional management practice often employs synthetic fertilizers and broad-spectrum pest- or herbicides, which can result in detrimental effects on bacterial evenness [35–37]. Albeit these negative effects on microbiota from conventional management, bacteria in the conventional agroecosystem site were still able to be reshaped by plants like those in the organic agroecosystem. Furthermore, a beta diversity analysis confirmed that crops grown in disrupted conventional soils showed significantly higher community dispersion compared the same crops growing in undisrupted conventional soils (p-value 0.015) suggesting that bacterial community alterations were mediated by plants following disruption in both conventional and organic soil.

The crops grown in the diseased soils were also able to recruit different rhizobacterial communities from one another (crop-effect p-value 0.001, R^2^: 0.148, Axis.2: 15.56%). However, disrupting the diseased soil did not allow any of the crops to recruit different bacteria from their same crop counterparts grown in the undisrupted soil (p-value: 0.080). A potential explanation of this could be based on the high presence of *Bacillus* and *Colstridium* genera, as these can survive heat treatment by the formation of thermotolerant endospores [38]. Thus, if these genera had survived autoclaving, they would likely dominate crop rhizo-communities when grown in the diseased soil. In our study, the relative abundance (RA) values of both *Bacillus* and *Clostridium* increased in abundance after autoclaving and crop growth, with the highest increase in RA being observed by *Bacillus* in the diseased agroecosystem.

### Crop-shared bacterial genera recruited following agroecosystem disruption

Differential abundance analyses revealed several bacterial abundance increases overlapping between different crops after growth across all disrupted soils (S10 Table). Eleven genera and two families (*Bosea*, *Caenimonas*, *Brevundimonas*, *Lacibacter*, *Luteolibacter*, *Pedobacter*, *Sphingoaurantiacus*, *Sphingopyxis*, *Dyadobacter*, *Larkinella*, *Rhabdobacter* and families Saccharimonadaceae and Sphingobacteriaceae; S10 Table) were observed to increase in abundance following disruption and growth of all four crops. We hypothesize that crops formed associations with some of these genera due to previous literature describing their plant growth promotional (or plant-symbiotic) nature. For example, members of *Bosea* and *Sphingopyxis* genera have been described to be PGPRs; and members of *Bosea* spp. can produce the auxin IAA [39, 40]. When *Dyadobacter* spp. were inoculated into soil, previous results showed the genus was positively correlated with nitrogen fixation and increased nitrate reductase activity in plant leaves [41]. Additionally, *Brevundimonas*, *Pedobacter*, *Luteoliobacter*, *Lacibacter*, and *Caenimonas* have all been isolated from the root communities of different plants [42–46]. Lastly, *Larkinella*, *Rhabdobacter* and *Sphingoaurantiacus* are genera that have been previously isolated from organic amendments (*Larkinella*) or soil systems (*Rhabdobacter and Sphingoaurantiacus*) [47–49].

Abundances of other bacterial genera were increased because of specific recruitment by three out of the four crops (but not all crops like those previously mentioned) following disruption, and these trends were also observed across pooled disrupted soils. The crop combinations that significantly increased the abundance of five bacterial genera (*Algoriphagus*, *Articibacter*, *Devosia*, *Oligoflexus*, *and Opitutus*) and additionally, these shared genera are reported to possess some potential PGP ability. Both *Opitutus* and *Devosia* genera were seen to be associated with rice rhizosphere [50, 51], and studies show that members of the *Devosia* genus possess a myriad of plant-benefiting functions (IAA synthesis, production of ammonia, and production of siderophores) [52, 53]. Additionally, the *Devosia* genus has been identified to comprise true plant-endophytes that colonize the interior tissues of tomato plants [54]. *Algoriphagus* has been observed to increase in relative abundance as a response to plant defense-inducing biochemicals (salicylic acid, methyl jasmonate, and abscisic acid) [55]. The notion that *Algoriphagus* shows increased abundance in response to plant-defense compounds may suggest an upregulation of plant-defenses carried out by members within the genus. Literature on *Articibacter* spp. are scant, but the genus is prevalent in soybean rhizosphere during the vegetative stage [56]. In addition, *Oligoflexus tunisiensis* was isolated from the rhizosphere of both buckwheat and barley [57].

### Crop-specific bacterial genera resulting from agroecosystem disruption

There were also observations of several potential PGPR genera specifically increased by individual crops across the pooled disrupted soils. The bacterial genera increased by beet were *Roseococus*, *Peredibacter*, *Flavisolibacter*, *Parasegetibacter*, *UTBCD1* and *Solimonas*. The genus *Roseococcus* falls within the family Acetobacteraceae which has been associated with nitrogen fixation and PGP-ability [58]. *Peredibacter* spp. are soil bacteria that are bacterivorous toward gram-negative bacteria, suggesting a biocontrol ability of pathogenic bacteria by members of the *Peredibacter* genus [59]. Other studies show increased relative abundances of the genus when tomato plants were inoculated with the PGPR *Pseudomonas* sp. RU47 [60]. *Flavisolibacter* is a genus positively correlated with disease suppression of *Rhizoctonia solani* [61]. The understudied *Parasegetibacter* and *UTBCD1* genera falls within the Chitinophagaceae family, and this family is comprised of genera recorded to possess plant-growth promotional ability [62, 63]. Lastly, members of *Solimonas* have been isolated from agricultural soils growing ginseng [64].

Corn growth significantly increased abundances of the genera *Sporocytophaga* and *Pseudomonas* in addition to the families Fibrobacteraceae and Rhodothermaceae. Of the promoted bacterial genera by corn, only *Pseudomonas* members have been extensively documented due to their plant growth promotional abilities [65–67]. However, the genus *Sporocytophaga* is widespread in soils and members such as *S*. *myxococcoides* can hydrolyze cellulose [68]. Additionally, the Rhodothermaceae family bacteria also possess cellulolytic and xylanolytic activity [69]. Likely both Rhodothermaceae and *Sporocytophaga* members aid soil ecosystem in cycling of plant detritus, exuding carbon sources for neighboring, potentially plant-benefiting microbes. Lastly, the family Fibrobacteraceae has been recorded to closely associate with wheat, which is in the same plant family as corn (Poaceae) [70].

Lettuce growth promoted the abundance of 17 bacterial reads, and four of the promoted genera have been associated with plant symbiotic or protective abilities (*Rhodoferax*, *Fluviicola*, *Cytophaga*, and *Knoellia)*. *Rhodoferax* members can degrade chemical herbicides [71], and *Fluviicola* is a common member of the rice rhizosphere [56]. Bacteria within the genus *Cytophaga* are present in the barley rhizosphere, and may contribute to the turnover of carbon, phosphorus, and nitrogen in soil ecosystems [72]. Lastly, a species within the *Knoellia* genus is a known endophyte of *Costus speciosus* (a type of ginger) [73].

Tomato growth in disrupted soils promoted nine bacterial genera (*Bacillus*, *Chelatococcus*, *Asticcacaulis*, *Yonghaparkia*, *Aminobacter*, *Cereibacter*, *Mucilaginibacter*, *Shimazuella*, and *Thermomonas*). *Bacillus* members are known genera documented on their plant growth promotional abilities [74–76], and *Bacillus* and *Asticcacaulis* were both considered members of the tomato endospheric bacterial community [77]. Two *Muciliginibacter* spp. were recently discovered to be PGPRs by increasing root length of tomato [78]. *Shimazuella* falls within the actinomycetes phylum and was isolated from *Pueraria candollei* (Kudzu) rhizosphere soil [79]. Another genus promoted by tomato, *Yonghaparkia*, can utilize the precursor to ethylene, ACC (1-Aminocyclopropane-1-carboxylic acid), as a nitrogen source [80]. *Aminobacter* members can produce the plant growth hormone cytokinin [81]. Lastly, some *Thermomonas* members are thermotolerant [82] explaining how these taxa were able to withstand disruption, although the literature on the relationship between genus and plants is lacking.

### Notable crop-shared bacterial genus-level abundance increases following soil disruption

Our data showed that several PGPR-related genera were significantly increased in abundance and associated with all crops tested when grown in soils following disruption. Accordingly, we speculate that plant rhizodeposition during early growth and development plays a strong contribution to fill “empty” niches (caused by disruption) with plant symbiotic and/or beneficial bacterial taxa. Since our experiment occurred during the first seven weeks of crop-growth, it is possible that some of these crop-shared genera may represent generalist-PGPRs, plant-symbiotic taxa, or bacterial keystone species (which are described as low abundance early colonizers that aid in the establishment of the plant’s core microbiome) [83–85].

While all crop-shared bacteria may not represent keystone species, instead these may indicate understudied or novel PGPRs/crop-symbiotic bacterial taxa. As an example, observations of *Luteolibacter* were of particular interest as this genus was increased by all four crop families (Poaceae, Amaranthaceae, Asteraceae and Solanaceae) after growth in disrupted agroecosystems. Notably, however, this genus was not increased in either NPCK (disrupted/undisrupted) which likely indicates a plant-symbiotic nature of *Luteolibacter*. Further the genus falls within the Verrucomicrobia phylum, a bacterial phylum known to be present in varying plant-soil ecosystem interactions [44, 84–86] in addition to being reported in the rhizospheres differing-family crops (Poaceae, Amaryllildaceae, and Solanaceae) [44]. Therefore, the bacterial taxa that showed an increased abundance and shared by all four crops may be worth investigating as novel and generalist PGPRs in further experimentation. We also suggest that they are responsive to plant presence (e.g. root exudation) as these bacterial taxa did not increase in abundance in the no plant controls.

### Conclusions

Our results indicate that bacterial responses to crop growth are amplified after soil disruption, and that crop-specific bacterial community changes were much weaker without the disruption treatment. Additionally, after disruption of the organic or conventional soils, taxa recruited in these soils significantly differed from taxa recruited by the same plants grown in the undisrupted organic or conventional soil, but this effect did not occur in the diseased soil. Additionally, results show that the combination of disruption and the introduction of a new crop to an agroecosystem is a promising means to achieve plant benefiting bacterial community rearrangement. Accordingly, the implementation of managerial practices to promote soil microbiome re-arrangement should be considered as we begin to re-generate agricultural soils. After disruption in all agroecosystems crops were shown to develop both crop-specific and crop-shared relationships alongside several bacterial genera. Crop-specific associations between these genera and their hosts may aid in future determination of core or symbiotic rhizobacteria for the plant species tested, whereas crop-shared taxa (e.g., *Luteolibacter*) may be of interest in future determination of novel and generalist PGPRs.

## Supporting information

S1 Fig Mean crop fresh-weight (FW) biomass (a) grown in organic soil (b) conventional soil and (c) diseased soil (n = 10 / treatment, ± standard deviation). Significant increases in biomass of each crop are denoted by “*”, “**” or “***” above each. Siddak’s multiple comparison test using GraphPad (Vers 8.2.1).
(TIF)

S1 Table Average Plant Fresh Weight (FW) Biomass and Percent Increases. (XLSX)

S2 Table Average Plant Dry Weight (DW) Biomass and Percent Increases. (XLSX)

S3 Table Pairwise comparison of qPCR results for values of 16s rRNA copies per g FW rhizosphere or bulk soil (NS = Non “sterile” and soils were not autoclaved before experimentation S = Sterile and soils were autoclaved before experimentation). (XLSX)

S4 Table Pairwise comparison between values of Shannon’s alpha diversity assigned to each treatment. (Above) Comparison of Shannon’s α-diversity values between disrupted and undisrupted treatments. (Below) Comparison of Shannon’s α-diversity values between communities detected in disrupted no plant treatments and disrupted plant treatments (e.g. Crop: Autoclaved = disrupted, Crop: Unautoclaved = undisrupted).
(XLSX)

S5 Table Bacterial genera observed to significantly increase in abundance after corn growth across all disrupted soils, compared to abundances of the same genera across undisrupted soils growing corn. If any abundance increases were observed from taxa listed in the NPCKs (S9 Table), they were removed from this table so included taxa may be attributed to corn presence. Unclassified genera increased in abundance after disruption are listed with their lowest classification level (k: kingdom, p: phylum, etc.) (FDR: False discovery rate).
(XLSX)

S6 Table Bacterial genera observed to significantly increase in abundance after beet growth across all disrupted soils, compared to abundances of the same genera across undisrupted soils growing beet. If any abundance increases were observed from taxa listed in the NPCKs (S9 Table), they were removed from this table so included taxa may be attributed to beet presence. Unclassified genera increased in abundance after disruption are listed with their lowest classification level (k: kingdom, p: phylum, etc.) (FDR: False discovery rate).
(XLSX)

S7 Table Bacterial genera observed to significantly increase in abundance after lettuce growth across all disrupted soils, compared to abundances of the same genera across undisrupted soils growing lettuce. If any abundance increases were observed from taxa listed in the NPCKs (S9 Table), they were removed from this table so included taxa may be attributed to lettuce presence. Unclassified genera increased in abundance after disruption are listed with their lowest classification level (k: kingdom, p: phylum, etc.) (FDR: False discovery rate).
(XLSX)

S8 Table Bacterial genera observed to significantly increase in abundance after tomato growth across all disrupted soils, compared to abundances of the same genera across undisrupted soils growing tomato. If any abundance increases were observed from taxa listed in the NPCKs (S9 Table), they were removed from this table so included taxa may be attributed to tomato presence. Unclassified genera increased in abundance after disruption are listed with their lowest classification level (k: kingdom, p: phylum, etc.) (FDR: False discovery rate).
(XLSX)

S9 Table Bacterial genera observed to significantly increase in abundance in the disrupted NPCKs, compared to abundance of the same genera across undisrupted NPCKs. Unclassified genera increased in abundance after disruption are listed with their lowest classification level (k: kingdom, p: phylum, etc.) (FDR: False discovery rate). Taxa listed in this S9 Table served as a control to filter out bacterial reads able to proliferate after disruption in the absence of plant growth.
(XLSX)

S10 Table Crop-specific and crop-shared bacterial abundance increases resulting from growth in disrupted agroecosystems. (XLSX)
