# Supplementary figures and images for: Soil microbiome disruption reveals specific and general plant-bacterial relationships in three agroecosystem soils
Source: PLoS One. 2022 Nov 16;17(11):e0277529. doi: 10.1371/journal.pone.0277529 (PMC9668122; doi:10.1371/journal.pone.0277529)

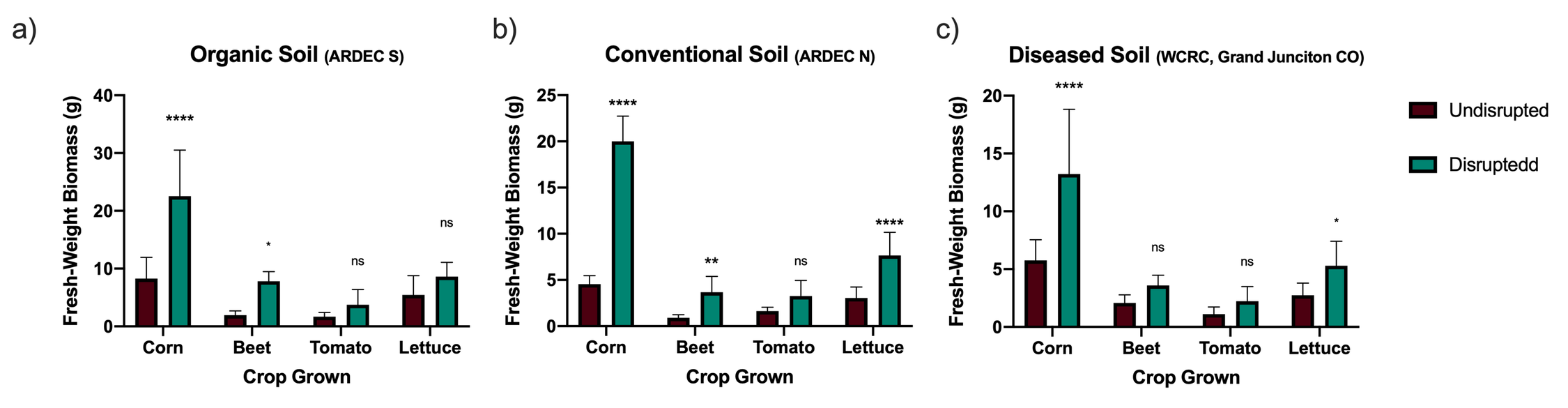

Supplement: S1 Fig — Significant increases in biomass of each crop are denoted by “*”, “**” or “***” above each. Siddak’s multiple comparison test using GraphPad (Vers 8.2.1). (TIF) [file pone.0277529.s001.tif]
